# Isoform Age - Splice Isoform Profiling Using Long-Read Technologies

**DOI:** 10.3389/fmolb.2021.711733

**Published:** 2021-08-02

**Authors:** Ricardo De Paoli-Iseppi, Josie Gleeson, Michael B. Clark

**Affiliations:** Centre for Stem Cell Systems, Department of Anatomy and Physiology, The University of Melbourne, Parkville, VIC, Australia

**Keywords:** isoform, long-read sequencing, PacBio, Oxford Nanopore Technologies nanopore sequencing, single cell sequencing, alternative splicing, spatial transcriptomics, targeted RNA sequencing

## Abstract

Alternative splicing (AS) of RNA is a key mechanism that results in the expression of multiple transcript isoforms from single genes and leads to an increase in the complexity of both the transcriptome and proteome. Regulation of AS is critical for the correct functioning of many biological pathways, while disruption of AS can be directly pathogenic in diseases such as cancer or cause risk for complex disorders. Current short-read sequencing technologies achieve high read depth but are limited in their ability to resolve complex isoforms. In this review we examine how long-read sequencing (LRS) technologies can address this challenge by covering the entire RNA sequence in a single read and thereby distinguish isoform changes that could impact RNA regulation or protein function. Coupling LRS with technologies such as single cell sequencing, targeted sequencing and spatial transcriptomics is producing a rapidly expanding suite of technological approaches to profile alternative splicing at the isoform level with unprecedented detail. In addition, integrating LRS with genotype now allows the impact of genetic variation on isoform expression to be determined. Recent results demonstrate the potential of these techniques to elucidate the landscape of splicing, including in tissues such as the brain where AS is particularly prevalent. Finally, we also discuss how AS can impact protein function, potentially leading to novel therapeutic targets for a range of diseases.

## Introduction

Alternative splicing (AS) enables the production of multiple RNA isoforms from single genes, greatly increasing both transcriptomic and proteomic diversity (Pan et al., 2008; Nilsen and Graveley, 2010). AS is the biological mechanism that controls which introns are removed from pre-mRNAs and which exons are joined to form the final messenger RNA (mRNA). Common AS events include skipped exons, retained introns and alternative 5′ and 3′ splice sites (Barbosa-Morais et al., 2012). It is now established that over 90% of multi-exon human genes undergo AS, and this prevalence highlights the biological importance of splicing events (Pan et al., 2008; Wang et al., 2008). Short-read sequencing approaches perform well at identifying AS of exons but are often unable to determine which full-length alternative isoforms are being expressed. The full extent of alternative isoform expression is only now starting to become clear with advances in long-read sequencing technologies that allow accurate determination of long-range exon connectivity (Sharon et al., 2013; Weirather et al., 2017).

The expression of different RNA isoforms can drive cellular differentiation, control cell functions and allow cells to respond to their environment (Roundtree and He, 2016). AS is highly regulated under normal conditions, while aberrant splicing contributes to various diseases including neurological disorders, autoimmune disorders and the development of cancer (Emilsson et al., 2008; Lee and Young, 2013; Sui et al., 2014; Vitting-Seerup and Sandelin, 2017). Splice-altering variants that cause disease are more prevalent than previously anticipated (Soukarieh et al., 2016; Rhine et al., 2018), and it has been predicted that one third of all disease-causing variants lead to aberrant splicing (Lim et al., 2011). The increasing estimates of disorders attributable to aberrant splicing highlights the need for technologies that can accurately detect these changes at the isoform level.

In this review, we discuss current and emerging long-read sequencing methodologies for full-length RNA isoform detection and quantification including target enrichment, single cell and spatial transcriptomics approaches and ask how these can help us uncover the roles of alternative isoforms in health and disease.

## Sequencing Technologies for Detecting RNA Isoforms

Early methods for transcriptome-wide identification of expressed genes and isoforms involved cloning cDNA libraries into vectors followed by Sanger sequencing to ascertain isoform sequences. While laborious, such methods, combined with innovations such as cap-trapping and normalisation, identified hundreds of thousands of full-length isoforms in various cells and tissues, helping to reveal the complexity of the transcriptome (Carninci et al., 1996; Strausberg et al., 2002). Conversely, short-read second generation sequencing of cDNA offers high-throughput, fast and affordable measurement of gene expression levels but with trade-offs for the accurate identification and characterisation of isoforms (Trapnell et al., 2010; Engström et al., 2013; Steijger et al., 2013). The current rising popularity of long-read third generation sequencing methods relates in-part to their potential to combine the advantages of previous sequencing methods and profile full-length isoforms quickly and affordably.

Short-read sequencing (SRS) with Illumina is currently the most popular sequencing technology. SRS is a well-supported method for transcriptomics and is both high-throughput and affordable (Mortazavi et al., 2008). Sequencing instruments including the MiSeq (Illumina) or Ion Torrent (ThermoFisher Scientific) can produce reads of up to 600 nt long, however reads are most commonly within the 100–200 nt range. While SRS captures information such as splice sites and transcription start and end sites, short read methods struggle to determine how these features are combined into isoforms due to the fragmentation of RNA prior to sequencing. Therefore, while short reads accurately quantify gene expression, they often fail to identify the correct isoform from which the read originates as isoforms from the same gene are largely similar (Steijger et al., 2013; Soneson et al., 2016; Zhang et al., 2017). Long-read sequencing (LRS) technologies commercialised by Pacific Biosciences (PacBio) and Oxford Nanopore Technologies (ONT) have a distinct advantage over short-reads as they can reliably generate reads that cover the entire isoform. This removes the challenging task of reconstructing possible transcript isoforms from fragmented short reads and can improve our understanding of alternatively spliced isoforms of complex genes (Chen et al., 2019; Clark et al., 2020).

Single molecule, real-time (SMRT) sequencing developed by PacBio (California, United States) detects differently labelled dNTPs as they are incorporated into a DNA strand (Eid et al., 2009). The read length when using SMRT sequencing is primarily limited by the longevity of the DNA polymerase, with average read lengths of >20 kb now possible (Hon et al., 2020). To achieve high accuracy, DNA molecules are circularised prior to sequencing, allowing circular consensus sequencing (CCS), where the polymerase progresses around the circularised template multiple times. This allows highly accurate consensus sequences (<1% error rate), also called HiFi reads, to be generated from a portion of individual subreads (Wenger et al., 2019). The PacBio Iso-Seq method uses SMRT sequencing to generate a set of full-length transcript isoforms (Gonzalez-Garay, 2016). Iso-Seq can be performed on either unamplified or PCR amplified cDNA to detect and quantify isoforms (Ambardar et al., 2016), though some protocols include size fractionation of cDNA and separate sequencing of the fractions, increasing detection of longer isoforms but limiting opportunities for isoform quantification.

Nanopore sequencing, commercialised by ONT (Oxford, United Kingdom), uses an array of biological nanopores within a membrane that translocate nucleic acid under an electric current (Deamer et al., 2016; Jain et al., 2016). As nucleotides pass through the pore, they cause characteristic disruptions in the current that allow for their identification by basecalling software (Deamer et al., 2016). Recent improvements to the basecalling software that converts the raw current signal into nucleotide sequence have brought nanopore read accuracies into a similar range to SMRT sequencing (∼90 to >99%) and both platforms continue to improve in this area (Wenger et al., 2019; Oxford Nanopore Technologies, 2021; Sahlin et al., 2021). Nanopore sequencing has no known length limit, with reads produced in excess of 2 Mb and length is primarily limited by the preparation and delivery of intact full-length sequences (Jain et al., 2016). Because sequencing is based on the detection of current changes caused by different nucleotides, nanopore sequencing can be performed on amplified and unamplified cDNA as well as native RNA (direct RNA sequencing). It can also detect epigenetic modifications, including RNA methylation, due to the characteristic current changes modified nucleotides create (Simpson et al., 2017; Lorenz et al., 2020).

Large numbers of reads are required to deeply profile the transcriptome and short-read methods will commonly generate 30–50 million reads per bulk sample. This is higher than the throughput currently obtainable with long-read platforms for the same experimental cost. However, PacBio can now generate up to 4 million HiFi reads on a Sequel II and ONT >50 million reads on a PromethION flow cell respectively. These continual increases in throughput have made comprehensive expression profiling feasible with long read platforms (Byrne et al., 2019).

Third generation, LRS technologies have been widely applied to discover and quantify gene isoforms across a range of species (including viruses, bacteria and plants), cell types and disease states. They have proved effective in characterising genes and isoforms in organisms with poorly annotated transcriptomes (Chen et al., 2017; Kim et al., 2019; Suryamohan et al., 2020), novel isoform discovery in well characterised organisms (Lagarde et al., 2017; Hardwick et al., 2019; Workman et al., 2019; Clark et al., 2020; Roach et al., 2020) and identifying changes in isoform profiles in disease (Asnani et al., 2020; Fujiyoshi et al., 2020; Tang et al., 2020). The portable nature of nanopore devices allows for use in the field and rapid response to emerging situations that can impact human health (Quick et al., 2015; Russell et al., 2018; Shaffer, 2019). Together these advantages are likely to see long-read methods continue to increase in popularity for expression and isoform profiling.

## Progress and Applications of Long-Read Sequencing for Profiling Spliced Isoforms

**FIGURE 1:**
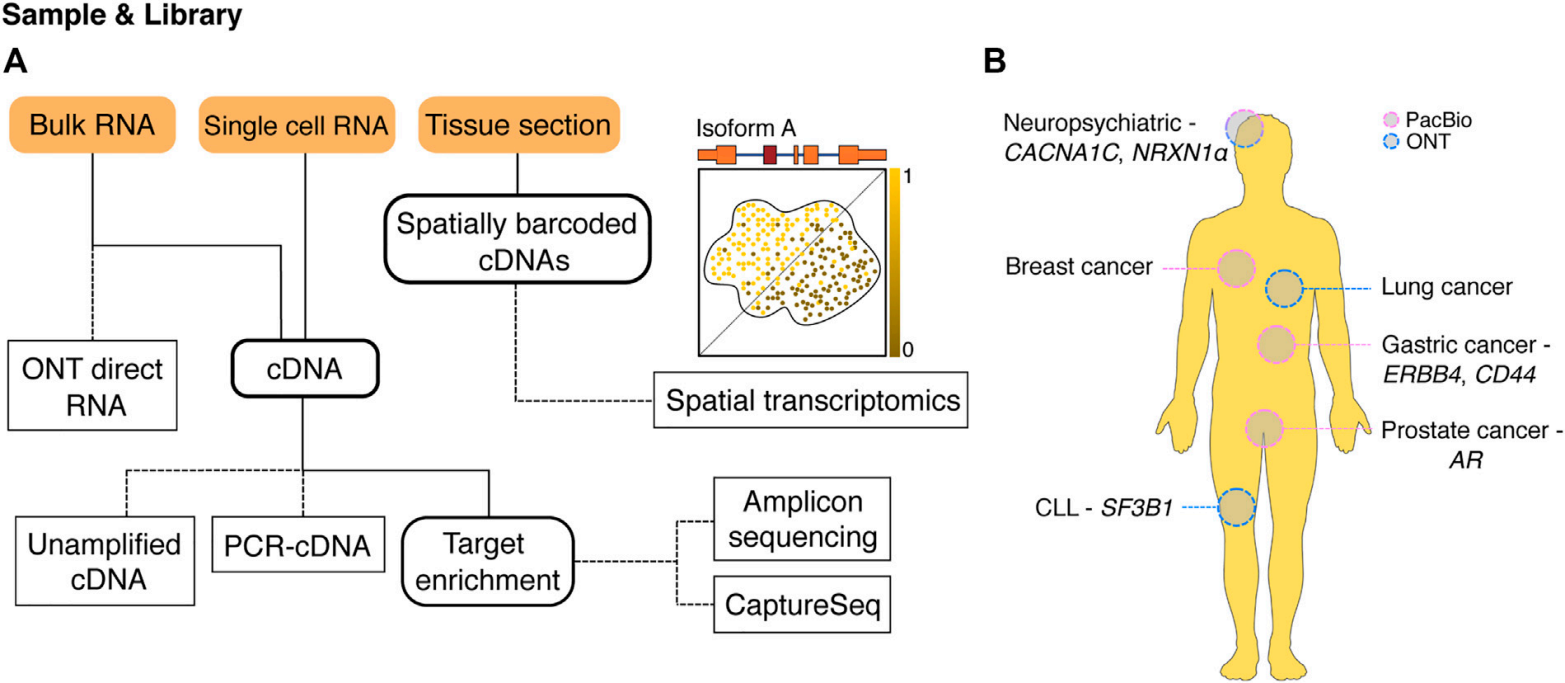
Long-read methods for profiling isoforms. **(A)** An overview of available long-read methodologies for isoform characterisation. A variety of sample inputs (orange boxes) can be used with different sequencing methods (dashed edges) to answer a wide range of experimental questions. Intermediate experiment steps are shown with a bold outline. RNA from bulk samples can be either sequenced directly (ONT direct RNA) or reverse transcribed to cDNA, while RNA from single cells currently needs to be processed into cDNA before sequencing. Once present as cDNA, samples can be sequenced directly without PCR (unamplified cDNA) or after whole-transcriptome PCR amplification (PCR-cDNA) depending on starting amounts. cDNA can also serve as input for target enrichment techniques including PCR for specific gene isoforms of interest (amplicon sequencing) or capture of target cDNAs for sequencing (CaptureSeq). Long-read spatial transcriptomics starts with tissue sections placed on slides covered with spatially barcoded oligonucleotides. Cellular RNA is captured by nearby barcodes allowing generation of spatially barcoded cDNAs. Sequencing identifies spatial expression patterns of RNA isoforms within the tissue such as the hypothetical isoform A. **(B)** Selected examples where long-read sequencing methods have been used to examine isoforms and alternative splicing in a range of human diseases. AR, androgen receptor; CLL, chronic lymphocytic leukemia; PacBio, Pacific Biosciences; ONT, Oxford Nanopore Technologies.

Long-read sequencing methods (Figure 1A) can cover entire transcripts within single reads, allowing for unambiguous identification of expressed gene isoforms. LRS can also be performed without any PCR or reverse transcription steps, reducing the amount of bias in expression quantification. These features allow for accurate quantification of both genes and isoforms, with results from LRS being comparable to or outperforming those from SRS (Oikonomopoulos et al., 2016; Garalde et al., 2018; Sessegolo et al., 2019; Soneson et al., 2019; Dong et al., 2020; Chen et al., 2021). Isoforms arising from genes with highly complex splicing patterns can be accurately detected with LRS, overcoming one of the major limitations of SRS (Clark et al., 2020; Chen et al., 2021).

In a recent benchmarking study, four sequencing methods (Illumina SRS, ONT: direct RNA, unamplified (direct) cDNA and PCR cDNA) were compared using five human cell lines (Chen et al., 2021). While SRS and LRS performed similarly for gene quantification, long-read methods outperformed Illumina for isoform level quantification. This was further highlighted in genes with a large number of similar isoforms where long-read quantification far exceeded short-read methods. Notably, there was a bias in the PCR cDNA data towards amplifying highly expressed genes, which was not observed in either the direct cDNA or direct RNA data. This suggested that although higher throughput can be achieved with amplification protocols, these may result in lower transcriptional diversity in the sequencing data. The authors found that thousands of genes were using multiple isoforms across the five cell lines. The most frequent difference between the major isoform and its alternatives was the use of alternative promoters, followed by exon skipping. The error rate of direct RNA sequencing is higher than in direct cDNA, however it was shown that isoform abundances were consistent across techniques, which indicated that the error rate is not a liability for isoform quantification. These results are consistent with those in a recent study using direct RNA sequencing that showed accurate isoform quantification and detection of differential isoform expression (DIE) in synthetic RNA spike-ins (Gleeson et al., 2020). Overall, the data presented in Chen et al. (2021) suggests that long reads improve transcriptome profiling compared to short reads, while also allowing for additional analyses that are only made possible with long-read sequencing.

Various computational programs have been developed to produce a set of high-confidence isoforms from LRS data such as FLAIR, FLAMES, SQANTI and TALON (Tardaguila et al., 2018; Wyman et al., 2019; Dong et al., 2020; Tang et al., 2020). These programs correct splice junctions within reads and collapse identical transcripts into a set of unique high-confidence isoforms. This set of isoforms can then be aligned against current genome annotations for the discovery of novel isoforms. As a result, many studies have begun to reveal the extent to which human genome annotations are still incomplete (Soneson et al., 2019; Workman et al., 2019; Gleeson et al., 2020; Tang et al., 2020; Glinos et al., 2021). The percentage of novel un-annotated isoforms found in these LRS studies is generally between 30 and 60%, further demonstrating how SRS misses many spliced isoforms. Although the false positive rate of novel isoform identification software remains unclear, studies on different cell lines have seen an overlap in the novel isoforms found (Glinos et al., 2021), and many novel isoforms have been validated with RT-PCR and Sanger sequencing (Tardaguila et al., 2018; Robinson et al., 2020; Uapinyoying et al., 2020). Additionally, these programs can utilise matched short-read splice junction information to inform splice junction correction and improve isoform quality (Tang et al., 2020). To further improve isoform detection and quantification, programs are now being developed specifically for estimation of isoform abundances from long-read data, such as NanoCount for direct RNA sequencing (Gleeson et al., 2020). Using knowledge of features unique to direct RNA, NanoCount improves quantification by more accurately identifying the isoform of origin for each read. Detection and quantification of isoforms with long reads has already been shown to outperform short reads, and this will only continue to improve as more tools are developed in this fast-paced field.

## Long Read Profiling of Splicing in Disease

Alternative splicing contributes to diseases such as neurological disorders, autoimmune disorders and cancers (Figure 1B) (Emilsson et al., 2008; Lee and Young, 2013; Sui et al., 2014; Vitting-Seerup and Sandelin, 2017). Alternative splicing has also been shown to regulate the immune response to inflammation (Bhatt et al., 2012), and recent investigation of DIE using long-read sequencing has revealed a conserved mechanism of alternative first exon usage following inflammation (Robinson et al., 2020). In total, 50 novel isoforms with alternative first exon usage were identified from mouse macrophages, including a dominant novel isoform from the well-studied interferon inducible gene *Aim2* (Robinson et al., 2020). Understanding the role of AS in the inflammatory response will be important to improve our understanding of the molecular mechanisms that control inflammatory-regulated genes and the development of autoimmune disorders.

LRS has also been used to characterise viruses including Varicella-zoster (VZV), which causes chickenpox and shingles, within infected human neuronal cells (Braspenning et al., 2020). VZV establishes lifelong latency in neurons and its reactivation causes chronic pain later in life (Solomon et al., 2014). LRS revealed the architecture of the VZV transcriptome finding high levels of transcriptional complexity and alternative splicing (Braspenning et al., 2020). The ability to characterise viral transcriptomes with long-read sequencing has been especially useful throughout the COVID19 pandemic. Nanopore sequencing of the SARS-CoV-2 virus revealed the dynamic nature of transcription during its replication cycle (Chang et al., 2021). As well as identifying differential expression of subgenomic mRNA (sgRNA) transcripts during infection, novel sgRNAs containing non-canonical splice junctions were found that may have a role in enhancing viral protein production (Chang et al., 2021). These studies focusing on viral infection highlight how long-read sequencing enables the detection of full-length isoforms to reveal underlying viral mechanisms and further insight into the viral replication cycle within host human cells.

Alternative splicing and isoform switching are involved in the development of many cancers (Kahraman et al., 2020), and these mechanisms also contribute to cancer treatment resistance (Sciarrillo et al., 2020). For example, specific splicing patterns confer resistance to the cancer T-cell therapy CART-19 used to treat leukemia (Shah et al., 2019). Alternative splicing of CD19 transcripts was shown to play a central role in resistance to CART-19 immunotherapies, which led to further investigation of these transcripts using ONT direct RNA sequencing of human B-cells (Asnani et al., 2020). The study confirmed that an intron retention event caused premature termination of the CD19 transcript and the ablation of protein expression. Mutations in the splicing factor *SF3B1* are known to impact alternative splicing in several cancers (Furney et al., 2013; Tang et al., 2020). Using ONT cDNA sequencing and FLAIR, Tang et al. (2020) generated full-length isoforms from chronic lymphocytic leukemia (CLL) blood samples. This method identified 35 alternative 3′ splice-sites and global downregulation of intron retention events in *SF3B1* mutant CLL compared to wild-type (Tang et al., 2020).

A recent study of ten gastric cancer cell lines using PacBio’s Iso-Seq technology identified approximately 39,000 novel isoforms, including for the known oncogenes *ERBB2* and *CD44*. Alternative promoters were frequently used in gastric cancer cells which often resulted in altered downstream CDS and 3′ UTRs (Huang et al., 2021). Full-length transcripts, using both ONT and PacBio reads, have also been sequenced to identify variants involved in treatment resistance in triple negative breast and lung cancer cell lines (Lian et al., 2019; Seki et al., 2019; Oka et al., 2021). Together, these studies highlight the need for long-read technologies to characterise cancer specific and/or cancer-causing isoforms with the potential to encode novel therapeutic targets or act as biomarkers (Kahles et al., 2018).

### Long-Read Profiling of RNA Processing

The widespread interdependency of transcription initiation and subsequent mRNA splicing and processing was recently established in human breast cancer cells using PacBio long-read sequencing, revealing that alternative transcription start sites had a significant impact on alternative splicing even across multiple exons and large distances (Anvar et al., 2018). These processes were tightly coupled in over 60% of genes with multiple transcripts and consistent across three other human cell types; brain, heart and liver. LRS is a promising method for investigating RNA regulation and processing, and these studies demonstrate our current incomplete understanding of coordinating mechanisms between transcription initiation and mRNA splicing.

### Characterising the Role of Genetic Variation on Isoform Expression

A recent study advanced long-read transcriptional profiling into the realms of population-scale analysis on a large number of individuals (Glinos et al., 2021). LRS of 88 Genotype-Tissue Expression (GTEX) samples discovered almost 100,000 novel human isoforms, including support for over 4,000 novel isoforms previously identified by Workman et al. (2019). Over half of the highly expressed novel isoforms were only expressed in a single tissue, highlighting a potential role for these isoforms in tissue specific functions. An allele-specific analysis was used to study the effects of common and rare genetic variants on both RNA expression and splicing. Splicing quantitative trait loci were shown to mostly result in exon skipping, while expression quantitative trait loci modified not only expression levels, but also changes to the 5′ end of transcript structures (Glinos et al., 2021). The authors highlighted the importance of studying the transcriptome at the level of isoforms and splicing rather than at the gene level, which is now possible with long-read technologies.

## Targeted Long-Read Sequencing

Long-read sequencing of whole transcriptomes has provided many insights into splicing and the role of RNA isoforms in health and disease. Expanding on these capabilities, a number of studies have now coupled LRS with technologies such as targeted sequencing of genes and isoforms of interest. Due to the wide dynamic range and often cell- or tissue-specific expression of RNAs, targeted methods are commonly needed for the detection and quantification of many isoforms (Mercer et al., 2014). This limitation to sequencing sensitivity affects LRS similarly to SRS and cannot easily be overcome with additional sequencing depth. RNA sequencing using CaptureSeq or PCR-amplicon sequencing has been successfully utilised to perform targeted long-read sequencing. These targeted approaches have been used to discover and annotate novel RNA isoforms, particularly of lowly expressed genes like long-noncoding RNAs (lncRNAs) (Lagarde et al., 2017; Hardwick et al., 2019); investigate splicing dynamics (Deveson et al., 2018); deeply profile disease gene isoform diversity and variation between tissues (Treutlein et al., 2014; Clark et al., 2020) and help confirm the impact of pathogenic variants on isoform expression (Helman et al., 2021). In this section we examine current targeted long-read sequencing methods and challenges in enriching for specific gene isoforms.

### RNA Capture Sequencing Using Long Reads

**FIGURE 2:**
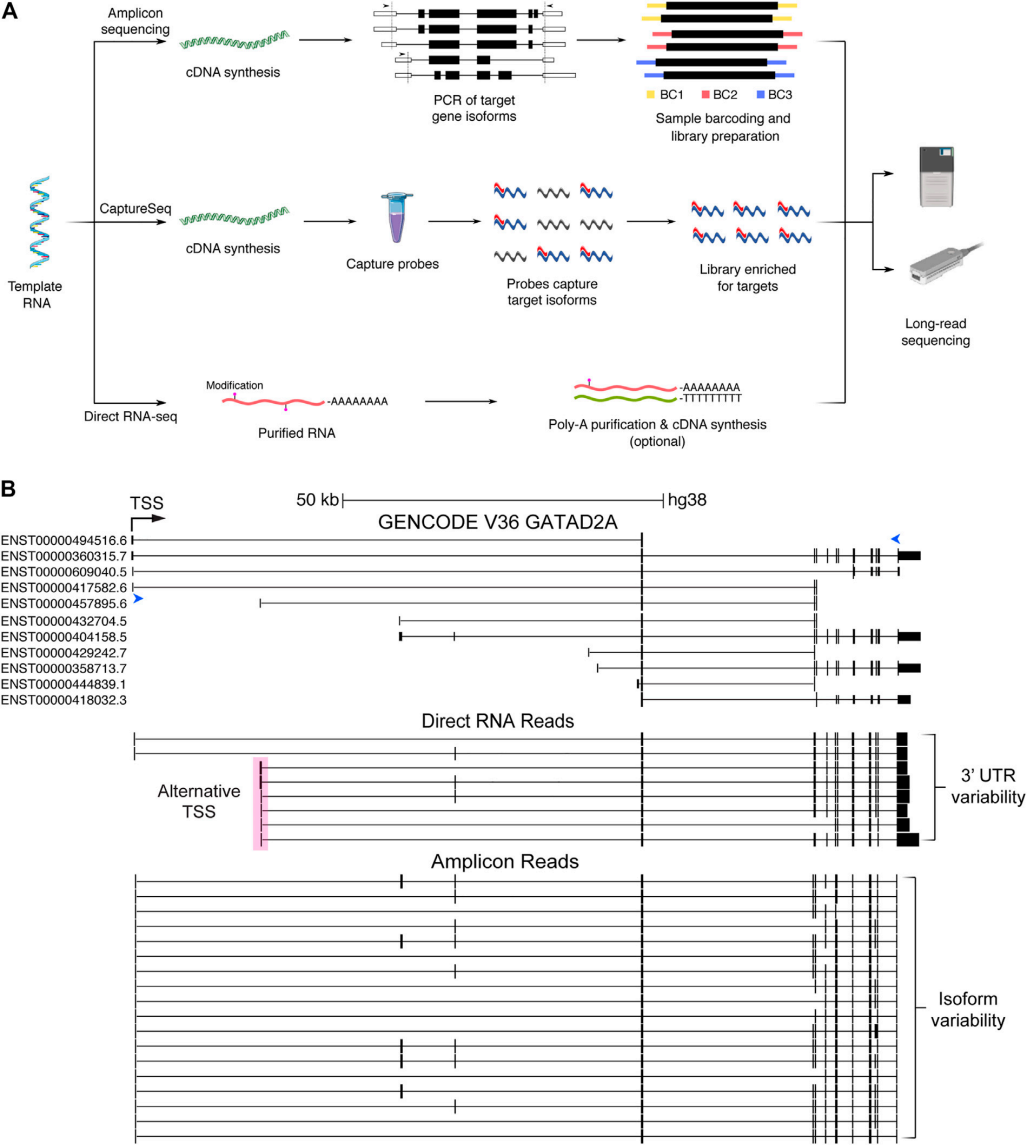
Long-read sequencing methods and data generated. **(A)** Simplified pathways describing three long-read sequencing methods: amplicon sequencing, CaptureSeq and direct RNA sequencing. Amplicon sequencing involves cDNA synthesis followed by PCR of known and novel expressed isoforms from target genes (arrows indicate locations of forward and reverse primers). Sample barcodes can additionally be used for multiplexing before sequencing. CaptureSeq is performed on cDNA and utilises pools of oligonucleotide probes (capture probes). Probes (red) hybridise to isoforms from target genes (blue), which can then be purified, creating a sequencing library highly enriched for target isoforms. Direct RNA sequencing requires purified RNA and commonly includes the optional preparation steps to purify polyA RNA (to remove ribosomal RNA) and perform cDNA synthesis (to break-up RNA secondary structures) respectively. Only the RNA strand is subsequently sequenced. **(B)** Schematic of isoform information generated by whole-transcriptome direct RNA sequencing vs gene-targeted amplicon sequencing of cDNA. Image from the UCSC Genome Browser of the schizophrenia risk gene *GATAD2A* (Ripke et al., 2014)*,* showing GENCODE annotations compared to nanopore direct RNA (middle) and amplicon (bottom) reads collapsed into high-confidence isoforms by FLAIR (Gleeson et al., 2020; Tang et al., 2020). Forward and reverse primers for amplicon sequencing are indicated by blue arrow heads. Transcriptional start site (TSS) shown by black arrow. An alternate start site (pink box) and 3′UTR variants are captured by direct RNA sequencing of the SH-SY5Y cell line. At least five supporting reads were required to identify high-confidence isoforms from direct RNA sequencing. A large number of additional isoforms are supported by amplicon sequencing using a more stringent threshold of 500 supporting reads. CDS, coding sequence; BC, barcode.

Despite concerted efforts using SRS, many expressed genes and isoforms remain unannotated or incorrectly assembled, particularly for complex genes with many isoforms or lowly expressed genes such as lncRNAs. RNA CaptureSeq uses oligonucleotide probes to enrich for transcripts from genes or genomic loci of interest (Figure 2A). CaptureSeq can enrich one to 1,000’s of genes allowing it to be applied to many gene sets of interest. Initially combined with SRS, it dramatically increased sequencing sensitivity, allowing for the discovery and quantification of new genes and isoforms (Mercer et al., 2014; Clark et al., 2015), however the identification of full-length isoforms was limited by the use of SRS.

Long-read RNA CaptureSeq (LCS) has now been successfully implemented in several studies to enable sensitive profiling of full-length isoforms. Lagarde et al. (2017) first developed LCS with PacBio sequencing to improve lncRNA annotations in human and mouse and thousands of novel full-length lncRNAs isoforms were discovered. The authors found that lncRNAs had at least twice as many isoforms as previously identified, and detection of lncRNA promoters and exonic structures was greatly improved (Lagarde et al., 2017). To expand the analysis of AS within lncRNAs, Deveson et al. (2018) used both short and long-read (PacBio) capture sequencing of human chr21, providing unprecedented sequencing depth to investigate transcription and splicing. Almost the entire non-repetitive length of chr21 was covered by spliced transcripts with very little exclusively intergenic space. Surprisingly, unlike coding transcripts which had defined alternatively spliced exons, almost every lncRNA exon could be alternatively spliced. The universal nature of AS for noncoding exons suggested lncRNA exons are modular in nature and that lncRNAs have a vast array of undiscovered alternative isoforms (Deveson et al., 2018).

Many genomic regions have been implicated in neurological disorders by genome-wide association studies (GWAS). However, many of these genomic regions are annotated as intergenic (Buniello et al., 2019). Short-read CaptureSeq has identified widespread noncoding transcription in these “intergenic” GWAS loci (Bartonicek et al., 2017). To further investigate this, long-read CaptureSeq with ONT was used to interrogate genomic regions implicated in neuropsychiatric disorders (Hardwick et al., 2019). LCS identified 109 novel high-confidence multi-exonic transcripts from these regions, along with new alternative isoforms of known brain genes, which were predicted to encode for novel protein variants (Hardwick et al., 2019). Gene and isoform discovery in “intergenic” GWAS regions will help facilitate functional genomic studies of how these regions contribute to disease.

A key limitation of CaptureSeq is the high cost of the oligonucleotide capture probes. Recently, Sheynkman et al. (2020) developed ORF CaptureSeq (OCS), a method to generate capture probes quickly and cheaply from cDNA clones. OCS performed similarly to commercially synthesised probe pools and the authors demonstrated the flexible implementation of OCS on between 2 and 763 human transcription factors. PacBio sequencing of 763 targeted transcription factors identified a 7-fold increase in isoforms compared to un-enriched samples. The development of OCS has the potential to broaden the applicability of CaptureSeq to a much wider range of studies (Sheynkman et al., 2020).

Generation of full-length isoforms using LCS takes a substantial step further towards a complete map of the human transcriptome, however there are some limitations that remain including the lower capture efficiency observed with long-reads and potential artifacts from incomplete reverse transcription, RNA degradation, or incorrectly identified splice junctions due to sequencing errors (Lagarde et al., 2017; Hardwick et al., 2019).

### Long-Read Amplicon Sequencing

Unlike transcriptome-wide approaches or CaptureSeq, amplicon sequencing approaches are lower throughput and usually applied to a small number of genes. A schematic overview of the differences between target PCR, CaptureSeq and direct RNA sequencing is shown in Figure 2A, with examples of the different outputs from these shown in Figure 2B. A relatively straightforward but powerful approach is to use long-range PCR, with primers in the 5′ and 3′ UTRs, to amplify full-length isoforms (or entire coding regions) of genes of interest. The advantages of amplicon sequencing are extremely deep profiling of target genes and better coverage of longer isoforms, which are underrepresented when amplifying a pool of transcripts of varying lengths. Early PacBio long-read amplicon sequencing in bovine (*Bos taurus*) resulted in approximately 50,000 full-length immunoglobulin G (IgG) cDNA reads and the characterisation of a similar number of variable antigen binding regions, which was previously not possible with SRS (Larsen et al., 2012). Nanopore amplicon sequencing was subsequently used to identify almost 8,000 isoforms of *Dscam1*, the most extensively alternatively spliced gene known in *Drosophila* (Bolisetty et al., 2015).

**FIGURE 3:**
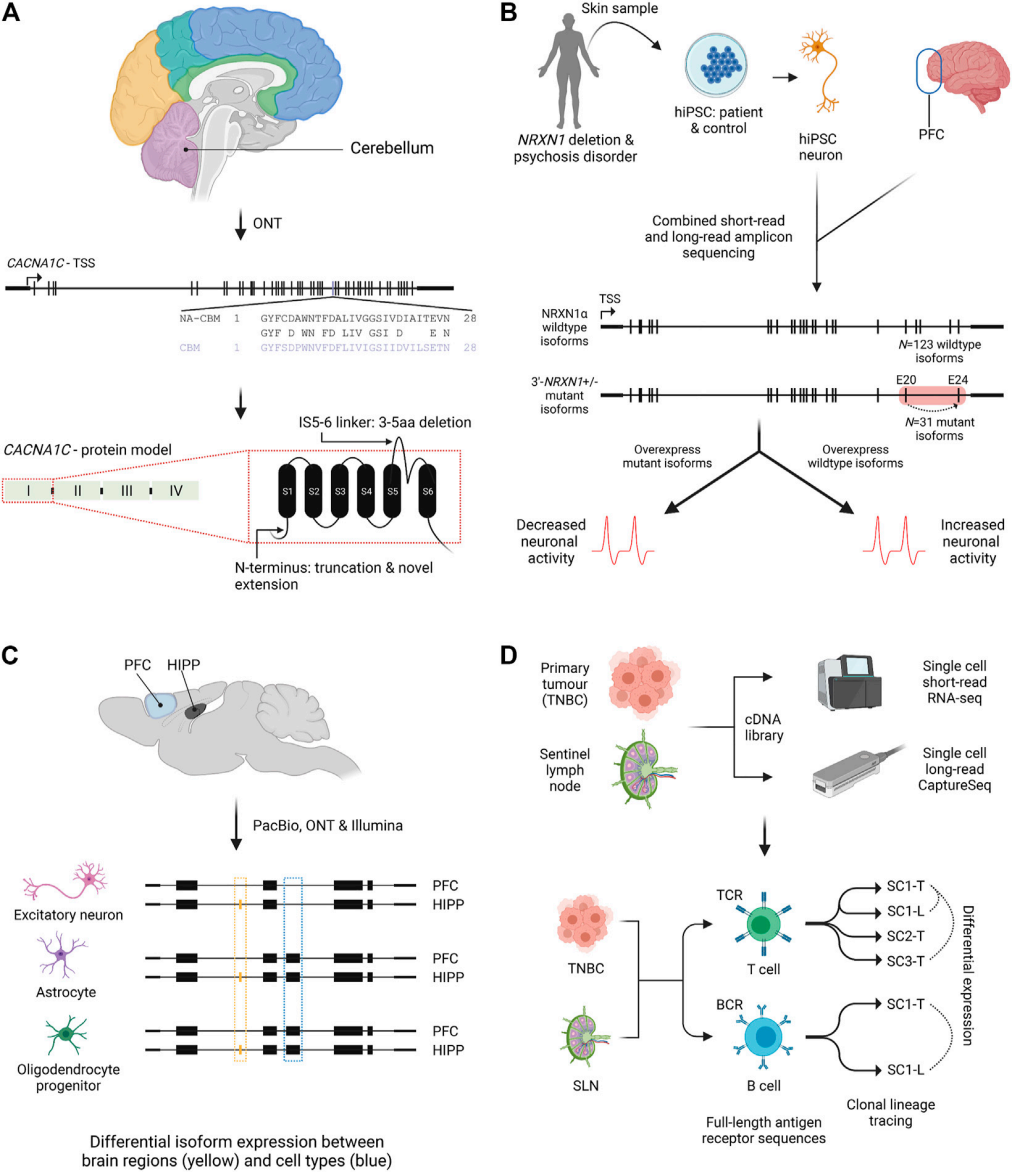
Isoform characterisation in health and disease. **(A)** Nanopore amplicon sequencing of *CACNA1C* in post-mortem human brain found an isoform switch in cerebellum (purple) for exon 30 compared to non-cerebellar tissues. An example of novel splicing impacts on the N-terminus and an extracellular region (IS5-6 linker) is also shown for the first *CACNA1C* protein domain (Clark et al., 2020). NA-CBM, non-cerebellar tissue; CBM, cerebellum; aa, amino acid. **(B)** Combined short and long-read amplicon sequencing of *NRXN1α* in PFC and *NRNX1* mutant and wildtype hiPSCs characterised expressed isoforms. Overexpression of mutant isoforms in wildtype hiPSCs or wildtype isoforms in mutant iPSCs identified genotype dependent impacts on neuronal activity. Red box indicates location of patient specific *NRXN1α* ^*+/-*^ deletions. Dashed arrow indicates exon skipping from exons 20 to 24 (Flaherty et al., 2019). PFC, prefrontal cortex; hiPSC, human induced pluripotent stem cell; TSS, transcriptional start site. **(C)** Single cell isoform RNA sequencing (ScISOrSeq) from mouse prefrontal cortex and hippocampus identified cell type signatures, e.g., exon exclusion (blue box) and tissue specific signatures e.g. exon inclusion in hippocampus (orange box) (Joglekar et al., 2021). PFC, prefrontal cortex; HIPP, hippocampus. **(D)** Repertoire and Gene Expression by sequencing (RAGE-Seq) characterised full-length antigen receptor sequences for T and B cells from a primary tumour (breast) and its sentinel lymph node. Clonal lineages and clonal expansions were identified, as well as differential expression between clonally expanded T or B cells from paired tumour and lymph node samples (Singh et al., 2019). TNBC, triple negative breast cancer; SLN, sentinel lymph node; TCR, T cell receptor; BCR, B cell receptor; SC-T, shared clonotype tumour; SC-L, shared clonotype lymph node; ONT, Oxford Nanopore Technologies; PacBio, Pacific Biosciences.

Changes in splicing can be important in imparting risk for complex disease, however the isoform repertoire of many disease genes and which isoforms play a role in disease remains poorly understood (Li et al., 2016). The calcium channel *CACNA1C* has been identified by GWAS as a risk gene for neuropsychiatric disorders such as schizophrenia (Ripke et al., 2014). Amplicon sequencing from six human brain regions identified 38 novel exons and 241 novel *CACNA1C* isoforms (Figure 3A). Nine of the ten most abundant isoforms were novel, and many were predicted to encode channels with altered functions (Clark et al., 2020). Similarly, investigation of another schizophrenia risk gene, *SNX19*, in post-mortem human brain identified a group of alternatively spliced isoforms missing the *SNX19* C-terminal protein domain. Upregulation of these isoforms, several of which were novel, was associated with disease risk (Ma et al., 2020).

Neurexins are critical for synapse formation and studies have suggested hundreds of different isoforms may be produced, however efforts to identify these were hampered due to the length of the transcripts (∼5 kb) (Ullrich et al., 1995). Treutlein et al. (2014) extensively profiled isoform diversity of three neurexin genes, *NRXN1*, *NRXN2* and *NRXN3* with PacBio amplicon sequencing in mouse prefrontal cortex (PFC). This unbiased approach identified between 9 and 258 isoforms per gene, the absence of any dominant isoform and the alternative splicing of an exon previously thought to be constitutive (Treutlein et al., 2014). A more recent hybrid approach performed both short and long-read amplicon sequencing to quantify *NRXN1α* isoforms in human PFC and in neurons derived from induced pluripotent stem cells (iPSCs) (Flaherty et al., 2019). A catalogue of 123 *NRXN1α* isoforms was identified, 86% of which showed conservation with the isoforms previously detected in mouse (Figure 3B). iPSC-neurons made from patients with *NRXN1α* mutations showed widespread isoform dysregulation, including loss of standard isoforms important for neuronal activity and expression of mutant isoforms inhibiting neuronal activity (Flaherty et al., 2019).

These studies on brain genes demonstrate that existing isoform annotations are far from complete and that novel transcripts may be playing a larger role in gene expression changes and in disease than previously understood. These findings can inform our understanding of the pathophysiology of complex neuropsychiatric disorders such as schizophrenia, where identifying the full repertoire of genes isoforms is a critical step towards a more genetically informed pathway to precision medicine (Treutlein et al., 2014; Flaherty et al., 2019). The identification of isoforms associated with disease risk, or disease-specific isoforms, also raises the possibility of creating isoform-specific therapeutics to target either the aberrant RNA or protein isoforms produced.

Long-read amplicon sequencing has also shown promise in contributing to the diagnosis of rare genetic diseases, especially those involving splicing changes. Long-read amplicon sequencing helped provide a molecular diagnosis for individuals with mitochondrial disease by confirming the inclusion of a cryptic exon in the mitochondrial Complex I subunit gene *NDUFB10*. This previously unannotated exon contained an early in-frame stop codon leading to nonsense mediated decay (NMD) of the altered transcript (Helman et al., 2021). This study highlighted the use of long-reads and multi-omic approaches to identify potential causes of disease where other genomic approaches have failed to determine a cause.

## Long-Read Sequencing for Single Cell Transcriptomics

Single cell RNA sequencing (scRNA-Seq) profiles gene expression in individual cells. Short-read (SR) scRNA-Seq is now well established and has been very successful in identifying cell types and trajectories; cellular and tumour heterogeneity; expression differences between cell types and for performing high-throughput perturbation assays (Hwang et al., 2018). SR scRNA-Seq methods separate into two main types, transcript counting methods that sequence only the 3′ or 5′ ends of transcripts (such as the popular 10x Genomics platform) and whole transcript methods that sequence reads from all regions of an RNA [such as Smart-Seq2 (Picelli et al., 2013)]. Transcript counting methods can profile large numbers of cells but provide limited splicing or isoform information, while whole transcript methods are lower throughput but provide more information on AS of exons. Single cell reads are typically tagged with unique molecular identifiers (UMIs - a unique sequence tag added to each molecule) and cell barcodes for accurate expression quantification and identification of the cell of origin respectively, information that needs to be measurable in any long-read (LR) scRNA-Seq method.

SR scRNA-Seq studies using “whole transcript” methods have identified significant cell-to-cell differences in isoform expression (Shalek et al., 2013; Marinov et al., 2014; Yap and Makeyev, 2016; Song et al., 2017). However, these studies largely focused on changes in the usage of specific exons or splice junctions due to short-read constraints, leaving the true complexity of AS and isoform expression within and between single cells unclear. The update to Smart-Seq3 (Hagemann-Jensen et al., 2020) incorporated UMIs into this methodology and improved isoform identification, however isoform reconstruction was poor beyond 1 kb and only ∼40% of molecules could be assigned to an isoform. LR scRNA-Seq can be integrated with both 10x and Smart-Seq style methods by omitting cDNA fragmentation steps (Gupta et al., 2018; Singh et al., 2019). Coupling long-reads with single cell sequencing can provide the currently missing isoform information and has the potential to once again revolutionise transcriptomics.

Initial studies combining long reads with single cell technology were all performed on less than ten cells using either ONT (Byrne et al., 2017) or PacBio (Macaulay et al., 2015; Karlsson and Linnarsson, 2017) sequencing. While the number of cells was low, Byrne et al. (2017) found hundreds of genes expressing multiple isoforms and DIE, while Karlsson and Linnarsson. (2017) results suggested that isoform diversity was an important source of biological variability between cells. These studies demonstrated the potential of incorporating long-read sequencing into single cell studies, with a number of LR scRNA-Seq studies on the 10x chromium platform now emerging.

The first demonstration of LR scRNA-Seq in a significant number of cells utilised the PacBio LRS ScISOr-Seq method, complemented by SR scRNA-Seq, to study neurons, astrocytes and microglia from mouse cerebellum (Gupta et al., 2018). While isoforms from over one thousand single cells were characterised, ScISOr-Seq only sequenced a small median number of reads (270) and genes (129) per cell. The ScISOr-Seq method was built upon more recently to investigate cell-type specific splicing across mouse brain regions (Figure 3C) (Joglekar et al., 2021). Deeper per-cell profiling identified DIE in 395 genes between mouse hippocampus and prefrontal cortex cells, including 76 high-confidence novel isoforms. Of the 395 genes, 36% exhibited differential transcription start or end sites, while 64% had splice-site usage differences. DIE between brain regions was largely due to a single cell type changing its isoform expression pattern, a critical insight into the relative importance of cell-types, brain regions and cell composition in defining splicing patterns (Joglekar et al., 2021).

The higher error rate of nanopore sequencing provides additional challenges for LR scRNA-Seq due to the critical importance of identifying the cell barcode and UMIs present in each read. To address this, Lebrigand et al. (2020a) developed ScNaUmi-Seq where both LR and SR scRNA-Seq were performed on single cells from the 10x platform. SR scRNA-Seq identified the cell barcodes and UMIs present facilitating their identification in the higher error nanopore data. ScNaUmi-Seq was highly accurate, however cell barcodes and UMIs were only identified in ∼30% of nanopore reads, suggesting significant room for future improvements through both increased read accuracy and enhanced identification algorithms. Applying ScNaUmi-Seq to over one thousand embryonic mouse brain cells identified a median of ∼2,500 genes per cell and very high correlations between nanopore and SR gene counts, which validated that LR data gave an accurate representation of the transcriptome (Lebrigand et al., 2020a). Differential isoform usage (DIU) between cell-types was observed for 76 genes, including pronounced isoform switching during neuronal development (Lebrigand et al., 2020a).

As demonstrated by Gupta et al. (2018), sequencing large numbers of cells with current LR scRNA-Seq technologies either results in low per-cell read depth or high experimental cost. This creates a trade-off between per-cell depth (important for isoform comparisons) and number of cells sequenced. FLT-Seq (Tian et al., 2020) is a recently developed method that subsamples 10–20% of the cells from a 10x Genomics experiment for nanopore sequencing, which combined with SR scRNA-Seq on all cells allows for an integrated analysis using the FLAMES package (Dong et al., 2020). SR scRNA-Seq identifies the cell barcodes and UMIs present, (similar to ScNaUmi-Seq) while also providing a broader view of the cell-types present. LR scRNA-Seq on over two thousand human and mouse cells generated a number of insights into AS and isoform usage at the single cell level, including that the two most abundant isoforms most commonly differ by multiple AS events and that isoform proportions are largely unimodal, not bimodal, in a cell population (Tian et al., 2020).

The previously mentioned studies using LR scRNA-Seq relied on short-reads in addition to long-reads for tasks including increasing the number of profiled cells, cell clustering, improving the accuracy in assigning a long-read to its cell of origin and correcting long-read splice junctions. In contrast, the higher accuracy R2C2 method was recently used to sequence full-length nanopore reads and provide isoform-level data from ∼1,500 blood cells without short-read support (Volden and Vollmers, 2020). The R2C2 method uses rolling circle amplification and concatemeric consensus sequencing to generate a read accuracy of 96% from nanopore data. SR sequencing was generated for comparison, confirming accurate gene quantification with R2C2. Along with establishing a short-read independent single cell method, a wide range of isoform diversity was found between genes, with high diversity genes like *LMNA* expressing a unique isoform in most cells (Volden and Vollmers, 2020). A trade-off of the R2C2 method is that by sequencing each cDNA multiple times for higher accuracy, the total number of unique cDNAs profiled is around 3x lower than standard methods. Additionally, 45% of R2C2 UMIs were unmatched in the SR data, demonstrating that even 96% accuracy is insufficient to assign many of the reads.

LR scRNA-Seq has also been combined with CaptureSeq to allow high sensitivity full-length profiling of isoforms of interest at single cell resolution (Singh et al., 2019). T-cell and B-cell receptors (*TCRs* and *BCRs*) are incredibly diverse and unique to each T- or B- cell lineage due to DNA rearrangement, AS and somatic hypermutation, however typing and tracing lineages at the single cell level has proven difficult (Picelli et al., 2013; Afik et al., 2017; Rizzetto et al., 2018). RAGE-Seq uses targeted capture of *TCR* and *BCR* isoforms followed by nanopore LR scRNA-Seq, coupled with short-read expression profiling of single cells (Singh et al., 2019). Applied to 7,138 cells from a tumour and lymph node of a breast cancer patient (Figure 3D), lymphocyte clonotypes and clonal expansions could be identified as well as differential expression between different T-cell clonotypes. One limitation was the low assignment of cell barcodes due to the error rate of nanopore sequencing, however, the capture-based method can be applied to any transcripts of interest, potentially overcoming the low-depth observed in other studies such as Gupta et al. (2018).

While the methodology of combining single cell and long-read technologies is still in its infancy, improvements are being made at a rapid pace. In future, as throughput and/or accuracy improves, subsampling and matched SR scRNA-Seq may become unnecessary. Single cell direct RNA sequencing may also become possible, allowing for single molecule isoform expression and modification profiling. The currently available studies highlight the relevance of analysing isoforms at single cell resolution, and we anticipate future research in the field to provide novel insights into the splicing landscape of cells and tissues.

## Long-Read Sequencing for Spatially Resolved Transcriptomics

While single cell sequencing has allowed us to uncover both known and novel cell types, the physical relationship between cells can now be studied with spatially resolved transcriptomics (Asp et al., 2020). Cells differentiate and function within tissues and are therefore influenced by their environment. Spatially resolved transcriptomics aims to profile gene expression patterns within this physical context. A large number of spatial methods have now been developed based on *in-situ* hybridisation, *in-situ* sequencing, or capture plus spatial barcoding of RNA (Asp et al., 2020).

The spatial transcriptomics method introduced by Ståhl et al. (2016) and now commercialised as the 10x Visium platform involves affixing tissue sections to slides covered with spatial barcodes allowing the generation of spatially barcoded cDNA. Two recent studies have adapted this platform for long-read sequencing. The first, spatial isoform transcriptomics (SiT), utilised nanopore long-reads combined with short-read sequencing for the assignment of UMIs and spatial barcodes (Lebrigand et al., 2020b). SiT was demonstrated in mouse brain, identifying 19 genes with isoform switching between regions in the olfactory bulb. For example, the *Plp1* gene involved in demyelination pathologies showed regional differences in isoform expression between the outer olfactory nerve layer and inner granule layer. In addition to spatial variation in isoform structure, SiT also identified spatial variation in isoform modifications. For example, A-to-I RNA editing events in the *Calm1* calcium receptor gene had robust variation between regions and showed a particularly high editing ratio in the thalamus (Lebrigand et al., 2020b). To investigate cell-type specific splicing and isoforms across mouse brain regions, Joglekar et al. (2021) developed slide-isoform sequencing (Sliso-Seq) to combine spatial transcriptomics with long-read sequencing. The author’s previous ScISOr-Seq technique had identified DIE of *Snap25* as neuroblasts matured into excitatory neurons. Sliso-Seq subsequently showed the switch occurred in a posterior-to-anterior gradient across the brain, enabling the linking of single cell results to the spatial dynamics of the *Snap25* isoform switch (Joglekar et al., 2021).

Applying long-read technologies to spatial transcriptomics will deepen our understanding of how alternative isoform usage and splicing influence cell processes. Currently the 10x spatial transcriptomics platform adapted by Lebrigand et al. (2020b) and Joglekar et al. (2021) does not provide single cell resolution. However, in the future, coupling long-read and single cell spatial transcriptomics methods will allow for the creation of three-dimensional maps of isoform expression from single cells. These insights will provide valuable knowledge into developmental mechanisms and pathways involved in disease.

## Remaining Challenges for Long-Read Sequencing

Long-read sequencing approaches excel at confirming exon connectivity and general transcript structure, however there are several limitations that users should be aware of when interpreting results. Amplification of target genes or whole transcriptomes carries the risk of PCR bias (shorter transcripts are favoured by PCR and will increase in proportion as more cycles are performed) and generation of chimeric cDNA (Brakenhoff et al., 1991; Bolisetty et al., 2015). Both issues can be mitigated by minimising PCR cycles, performing direct cDNA or RNA sequencing or through the use of UMIs (Lebrigand et al., 2020a; Karst et al., 2021). UMIs proved particularly useful for identifying robust differential isoform usage (DIU) in genes that appeared to have consistent expression between conditions (Lebrigand et al., 2020a). UMIs can also be used to reduce the relatively high error rates from nanopore sequencing by clustering together multiple reads from an original sequence (Karst et al., 2021).

Sample quality is critical for long-read data as degraded RNA or DNA can lead to fragmented sequences negating the primary advantage of long-read approaches. The quality of RNA, measured by the RNA integrity number (RIN), can vary significantly between samples and sample types. A minimum RIN of 6 (optimally > 7), was recommended for long-read amplicon sequencing (Clark et al., 2020), however the necessary RNA integrity for unbiased transcriptome-wide sequencing could potentially be higher. Additionally, post-mortem tissues are commonly used for human transcriptome analysis and the impact of death, post-mortem interval and ischemia should be considered when interpreting gene expression data (Ferreira et al., 2018).

The accuracy of ONT and PacBio long reads is generally lower than that of SRS and this can impact determination of splice sites and isoform identity as well as barcode and UMI assignment (Amarasinghe et al., 2020). In addition to the error rate, long-read methods do not always generate full-length reads due to compromised sample quality, library preparation limitations and incompletely sequenced reads. This further complicates isoform identification and quantification as reads can have multiple transcriptome alignments. Therefore, correctly identifying the isoform of origin is still a challenging task (Sessegolo et al., 2019; Soneson et al., 2019). Error correction and isoform identification tools, such as SQANTI and FLAIR, correct reads using long-read consensus or paired SRS data (Tardaguila et al., 2018; Tang et al., 2020). These tools address truncated reads by collapsing partial isoforms into the longer isoforms they likely represent and setting a minimum read threshold for an isoform to be considered valid. Benchmarking of these tools and the details of different error correction approaches and novel isoform assignment has been reviewed in detail elsewhere (Zhang et al., 2017; Amarasinghe et al., 2020), however false positive isoform identification remains a challenge. While software improvements are an avenue for improvement, isoform identification will also improve with decreased errors rates and sample preparation techniques that lead to less truncated reads (Amarasinghe et al., 2020).

## The Impact of Alternative Splicing on Protein Function and Abundance

As long-read sequencing continues to build a foundation of high-confidence isoforms, particularly for complex genes, the question of how these isoform changes functionally impact the proteome can begin to be answered. There is considerable debate over functional roles for alternative transcripts, with some studies reporting a single major isoform for most genes and little evidence for protein variants due to alternative splicing (Djebali et al., 2012; Gonzàlez-Porta et al., 2013; Tress et al., 2017). Other studies have shown evidence to the contrary, including the Vertebrate Alternative Splicing and Transcription Database (VastDB), which reported pronounced isoform switching in 48% of multi-exonic human genes and multiple co-expressed major isoforms in 18.5% (Tapial et al., 2017). Up to 75% of isoforms with exon-skipping have also been reported to be engaged by the ribosome, suggesting many are translated (Weatheritt et al., 2016). In addition, a splicing perturbation study by Liu et al. (2017) demonstrated that changes in RNA isoform expression led to changes in protein expression. More broadly, alternative isoforms such as non-coding isoforms, NMD isoforms or isoforms with altered UTR sequences can regulate gene expression and function even if the proteins produced do not change. Some of the debate may be down to perspective, many genes do appear to have a single dominant isoform in a cell type, however this does not mean other isoforms are not functionally relevant or become more prominent in other tissues or developmental timepoints, for which there is also plentiful evidence (Lebrigand et al., 2020a; Lebrigand et al., 2020b). In contrast, other genes have extremely complex isoform expression patterns and are poorly annotated by prior methods (Clark et al., 2020). Therefore, our understanding of the functional impact of alternative isoforms is constantly evolving (Weatheritt et al., 2016; Ule and Blencowe, 2019).

In their targeted study of the calcium channel gene *CACNA1C*, Clark et al. (2020) predicted the impact of novel full-length RNA isoforms on protein function. They found 51/83 (∼61%) of novel high-confidence isoforms detected using nanopore sequencing potentially encoded a functional Ca^2+^ channel. An additional splice-site-level analysis identified almost half of the isoforms had splice junction changes encoding microdeletions of 3–5 amino acids within regions previously implicated in channel conductance (Clark et al., 2020). Such results will be important to follow up. Several computational approaches are available to predict the impact of alternative RNA isoforms on protein features. For example, protein 3D structure and solubility can be predicted with programs such as Alternative Splicing-induced ALteration of Protein Structure (AS-ALPS) (Shionyu et al., 2009), DeepGOPlus (Kulmanov and Hoehndorf, 2019), PaRSnIP (Rawi et al., 2018), GOLabeler (You et al., 2018) and Deep Splicing Code (DSC) (Louadi et al., 2019).

Whilst computational predictive and simulation-based approaches are useful, validation using established and emerging proteomic technologies is the gold standard for assessing any biological impact of alternative mRNA isoforms on the proteome. Like RNA sequencing, proteomic methods have their caveats, identifying novel sequences and protein isoforms is generally more difficult with mass spectrometry than for transcriptomics (Blencowe, 2017). In addition, understanding the relative detection sensitivity of each method will be essential for successfully quantifying the relationship between RNA and protein isoforms. Thanks to recent advances, integration of genomics, transcriptomics and proteomics to improve our understanding of traits and diseases is now feasible, opening up new opportunities to examine the role of RNA and protein isoforms in health and disease (Molendijk and Parker, 2021).

## Discussion

Long-read sequencing enables profiling of full-length RNA and cDNA reads, which is essential for mapping alternative RNA isoforms in tissues and disease states. Coupling long-read data with both short reads and cutting-edge technologies such as single cell sequencing significantly widens the toolset for accurate isoform discovery in complex transcriptomes. Long read methods currently involve a trade-off between higher accuracy (Hifi, R2C2) and higher throughput (Nanopore, PacBio subreads). While lower accuracy can necessitate sophisticated error correction tools and/or paired short-read data, the advantages of long-over short-reads for isoform detection and quantification already outweigh many of the drawbacks in error rate (Chen et al., 2021). Furthermore, we anticipate that error correction will not be necessary in future due to the rapid pace at which long-read technologies are improving.

Long-read transcriptomics is still a nascent field of research, however it has already had a major impact on our understanding of spliced isoform diversity and expression (Lebrigand et al., 2020a; Clark et al., 2020; Joglekar et al., 2021). As transcriptomics moves into the age of the isoform, long-read technologies that enable transcriptomic characterisation at single cell and spatial resolutions will lead the way for new discoveries in health, development and disease.

## Glossary

5mC: 5-methylcytosine
AS: alternative splicing
BCR: B-cell receptor
CLL: chronic lymphocytic leukemia
CCS: circular consensus sequencing
CDS: coding sequence
DIE: differential isoform expression
DIU: differential isoform usage
GWAS: genome-wide association study
GTEX: genotype-tissue expression
iPSC: induced pluripotent stem cell
lncRNAs: long-noncoding RNAs
LCS: long-read RNA CaptureSeq
LRS: long-read sequencing
LR scRNA-Seq: long-read single cell sequencing
m6A: N6-methyladenosine
NMD: nonsense-mediated decay
OCS: ORF Capture-Seq
ONT: Oxford Nanopore Technology
PacBio: Pacific Biosciences
R2C2: Rolling Circle Amplification to Concatemeric Consensus
SiT: Spatial Isoform Transcriptomics
SRS: short-read sequencing
SR scRNA-Seq: short-read single cell sequencing
SMRT: single molecule, real-time
scRNA-Seq: single cell sequencing
sgRNA: subgenomic RNA
TCR: T-cell receptor
TSS: transcription start site
UMI: unique molecular identifier
UCSC: University of California Santa Cruz
UTR: untranslated region
VZV: varicella-zoster virus
VastDB: vertebrate alternative splicing and transcription database
