# Corrigendum: Isoform Age - Splice Isoform Profiling Using Long-Read Technologies

**DOI:** 10.3389/fmolb.2021.767743

**Published:** 2021-09-27

**Authors:** Ricardo De Paoli-Iseppi, Josie Gleeson, Michael B. Clark

**Affiliations:** Centre for Stem Cell Systems, Department of Anatomy and Physiology, The University of Melbourne, Parkville, VIC, Australia

**Keywords:** isoform, long-read sequencing, PacBio, Oxford Nanopore Technologies nanopore sequencing, single cell sequencing, alternative splicing, spatial transcriptomics, targeted RNA sequencing

In the original article, the reference for Kahraman et al. (2020) was incorrectly written as “Kahraman, A., Karakulak, T., Szklarczyk, D., von Mering, C., Kahraman, A., Karakulak, T., et al. (2020). Pathogenic Impact of Transcript Isoform Switching in 1,209 Cancer Samples Covering 27 Cancer Types Using an Isoform-specific Interaction Network. Sci. Rep. 10, 14453. doi:10.1038/s41598-020-71221-5.”

The correct reference is “Kahraman, A., Karakulak, T., Szklarczyk, D., and von Mering, C. (2020). Pathogenic Impact of Transcript Isoform Switching in 1,209 Cancer Samples Covering 27 Cancer Types Using an Isoform-specific Interaction Network. Sci. Rep. 10, 14453. doi:10.1038/s41598-020-71221-5.”

Additionally, there was an error in the **Discussion** section, which meant one sentence did not convey the intended meaning. A correction has been made to **Discussion**, **Paragraph 1**:

“Long-read sequencing enables profiling of full-length RNA and cDNA reads, which is essential for mapping alternative RNA isoforms in tissues and disease states. Coupling long-read data with both short reads and cutting-edge technologies such as single cell sequencing significantly widens the toolset for accurate isoform discovery in complex transcriptomes. Long read methods currently involve a trade-off between higher accuracy (Hifi, R2C2) and higher throughput (Nanopore, PacBio subreads). While lower accuracy can necessitate sophisticated error correction tools and/or paired short-read data, the advantages of long-over short-reads for isoform detection and quantification already outweigh many of the drawbacks in error rate (Chen et al., 2021). Furthermore, we anticipate that error correction will not be necessary in future due to the rapid pace at which long-read technologies are improving.”

The authors apologize for these errors and state that they do not change the scientific conclusions of the article in any way. The original article has been updated.
